# Trueness of tooth modified scan bodies as a novel technique for edentulous full arch implant supported dental prosthesis: an in vivo prospective comparative study

**DOI:** 10.1186/s12903-024-05172-y

**Published:** 2025-01-06

**Authors:** Abdelrahman K. Eldabe, Doaa Adel-Khattab, Kirollos H. Botros

**Affiliations:** 1 Oral Medicine, Periodontology and Oral Diagnosis, Faculty of Dentistry, Assiut University, Assiut, Egypt; 2 Oral Medicine, Periodontology and Oral Diagnosis, Faculty of Dentistry, Ain Shams University, Cairo, Egypt

**Keywords:** Scan body, Digital impression, Intra-oral scanning, Full arch, Novel scan body

## Abstract

**Objective:**

This study aimed to evaluate the clinical performance (degree of trueness) of a novel scan body “tooth-modified Scan body” (TMSB)& conventional scan body (CSB) in implant-supported full arch screw retained cases.

**Methods:**

Seven edentulous arches (two maxillae, five mandibles) in 6 patients were rehabilitated with monolithic zirconia screw-retained implant prostheses supported by 4 (*n* = 1) and 5 implants (*n* = 6) for a total amount of 34 implants. Implant locations were scanned by intra-oral scanner (IOS) using two types of scan bodies, conventional scan bodies (CSB) in group (1) and tooth-modified scan bodies (TMSB) in group (2). 68 implant positions (representing the total sample size) were captured and compared to the relative reference scans regarding angular and Euclidian deviation (ΔEUC).

**Results:**

The ΔEUC deviation Of TMSB group had lower values (M = 61.46, SD = 42.12) than that Of CSB group (M = 97.97, SD = 56.69). This difference was statistically significant (*p* = .005), 95% Confidence interval. The angular deviation of TMSC group had lower values (M = 0.85, SD = 0.69) than that Of CSB group (M = 1.3, SD = 1.06) which was statistically significant (*p* = .033), 95% Confidence interval. There was a correlation between the type of Jaw and both angular and ΔEUC deviation of both groups, which was statistically significant.

**Conclusion:**

A tooth-modified scan body (TMSB) may improve the ease and trueness of full-arch implant scanning, even in challenging mandibular arches.

**Clinical trial registry date:**

Retrospectively registered in 20/ 12/ 2023.

**Clinical trial registry number:**

NCT06177782.

## Introduction

Intra-oral optical impression through the use of intra-oral scanners (IOSs) can be considered a valid alternative to conventional impression materials when registering intraoral structures and implant position, improving the accuracy, predictability, and comfort of the patient [1–3]. Conventional impression materials and techniques have been thoroughly studied in the literature [4]. An incorrect transfer of the implant location can result in the creation of a subpar prosthesis, which can cause difficulties in delivery, less satisfaction to the patient, and subsequent complications [5, 6]. It has been demonstrated that taking a final impression with splinted impression copings produced better outcomes and a more accurate cast in patients with partial or total edentulism than taking impressions without splinting them [7, 8].

With the development of computer-aided design/computer-aided manufacturing; (CAD-CAM) technology, it is possible to skip a lot of conventional steps and fabricate dental prostheses through a more predictable workflow. Direct scanning intra-orally provides various benefits, such as better patient comfort, quicker scanning, and lower storage and transport requirements. Utilizing IOSs in implant impression involves the use of intra-oral scan bodies (ISBs) as digital fixture locators [9, 10].

An implant-supported fixed dental prosthesis (FDP) perfect fit was correlated with impression accuracy. Accuracy is defined by the International Organization for Standardization (ISO) as a combination of trueness and precision. While trueness is the degree to which measurements come close to the actual value, precision evaluates the variation and repeatability of the measurements. A scanner with high trueness creates a three-dimensional (3D) reproduction that closely resembles the scanned object. On the other hand, a scanner with great precision produces measurements with more consistent findings after repeated measurements [11].

IOSs capture single, consecutive images, which are then combined and realigned using the processing software and a best-fit alignment method. If present, anatomical features like teeth facilitate the “stitching” technique used to process images [8]. IOSs revealed a satisfactory accuracy in cases involving dentate patients that were comparable to polyether impressions and superior to alginate impressions [12]. However, a variety of factors, including saliva, tongue and cheek movements, the amount of attached gingiva, the length and shape of the edentulous ridge, the number and location of the implants, the inter-implant distance and angulation of the implants, as well as the characteristics of the ISBs and IOSs, can adversely affect the performance of IOSs particularly in complete arch scanning [3, 13].

Regarding the utilization of IOSs in capturing single implant, and quadrant implant cases, the mainstream of studies revealed a high degree of trueness that is usually ≤ 50 microns which is considered a clinically acceptable range of misfit [14, 15]. A discrepancy up to 150 mm has been widely adopted as the clinically acceptable level of misfit of implant-supported prostheses [16, 17]. IOSs in full arch implant cases remain a challenge for clinicians due to the lack of stable landmarks, and most studies reveal an unacceptable passivity, especially in lower arch cases [14, 18]. Therefore, conventional splinted open-tray impressions remain the standard impression method for complete-arch implant-supported prostheses [6].

Some authors have suggested maneuvers to boost the precision of a scan of a fully edentulous arch in order to get around these restrictions [19, 20]. An auxiliary geometric part (AGP) simulating a jaw with teeth was used in an in vitro study, thus filling in the gaps between the scannable impression copings. The authors achieved a significant improvement in both the trueness and precision of the complete-arch digital impression of the edentulous maxilla, which also facilitated the scanning procedure [2].

Since the absolute majority of these findings are reported by in vitro studies and using an auxiliary geometric part (AGP), especially when simulating teeth, remarkably improves the accuracy in full arch implant cases, the aim of this in vivo study is to evaluate the clinical performance of a novel scan body (Tooth Modified Scan Body) (TMSB) in the degree of trueness of IOS impression for full arch cases. TMSB is a scan body to which different teeth are attached directly as one piece. This may enhance the accuracy level by filling the gaps between intra-oral scan bodies (ISB) thus acting as stable, artificial intelligence (AI) easily recognized landmarks thus facilitating the scanning procedure. To our knowledge, it is the first time in the literature to utilize a novel scan body modified with directly connected teeth as artificial landmarks in vivo.

The null hypothesis was that both tooth-modified scan bodies and conventional scan bodies would show an equivalent degree of trueness. The primary outcome was to evaluate the clinical performance of tooth-modified scan bodies (TMSB)& conventional scan bodies for each patient enrolled in the study with a paired comparison of the deviation differences (degree of trueness). Secondary outcomes were to analyze the potential effect (correlation) of the arch type (maxilla vs. mandible) on the degree of trueness. In addition, the scanning time of the 2 techniques were compared.

## Methods

### Patients recruitment

All patients were recruited at the periodontology clinic of the Faculty of Dentistry Ain Shams University and Assiut University. The inclusion criteria were participants who had received 4–6 implants in 1 edentulous arch, who would receive 1-piece implant-supported complete-arch fixed dental prostheses, who would be available to attend follow-up visits, and who were medically healthy (American Society for Anesthesiology classification I or II). Multiunit abutments were installed at the time of implant placement (Neobiotech, Seoul, Korea). The implant stability quotient was measured, with a minimum value of 70 ISQ scale. All included participants would sign an informed consent.

### Sample size calculation

The Sample Size was calculated based on the primary outcome measure which is the Euclidean distance (ΔEUC). According to a previous study reporting a standard deviation of 115.5 and a mean difference of 49.6, calculations based on a dependent t-test with a power = 95% and a significance level *p* = .05 yielded a total sample size of 57 [18]. The number was increased to 60 to account for any possible dropouts. The adequacy of the sample size used was determined using Ps Power and Sample Size software (version 3.1.2). The unit of the sample size is the captured implant position, not the number of patients or the number of implants. Therefore, Implants in the same patients (a single cohort comparative study) were scanned twice by an intra-oral scanner (IOS) using two types of scan bodies, conventional scan bodies (CSB) in group (1) and tooth-modified scan bodies (TMSB) in group (2). 68 implant positions (representing the total sample size) were captured and compared to the relative reference scans regarding angular and Euclidian deviation (ΔEUC).

### Clinical and laboratory protocol

**Fig. 1:**
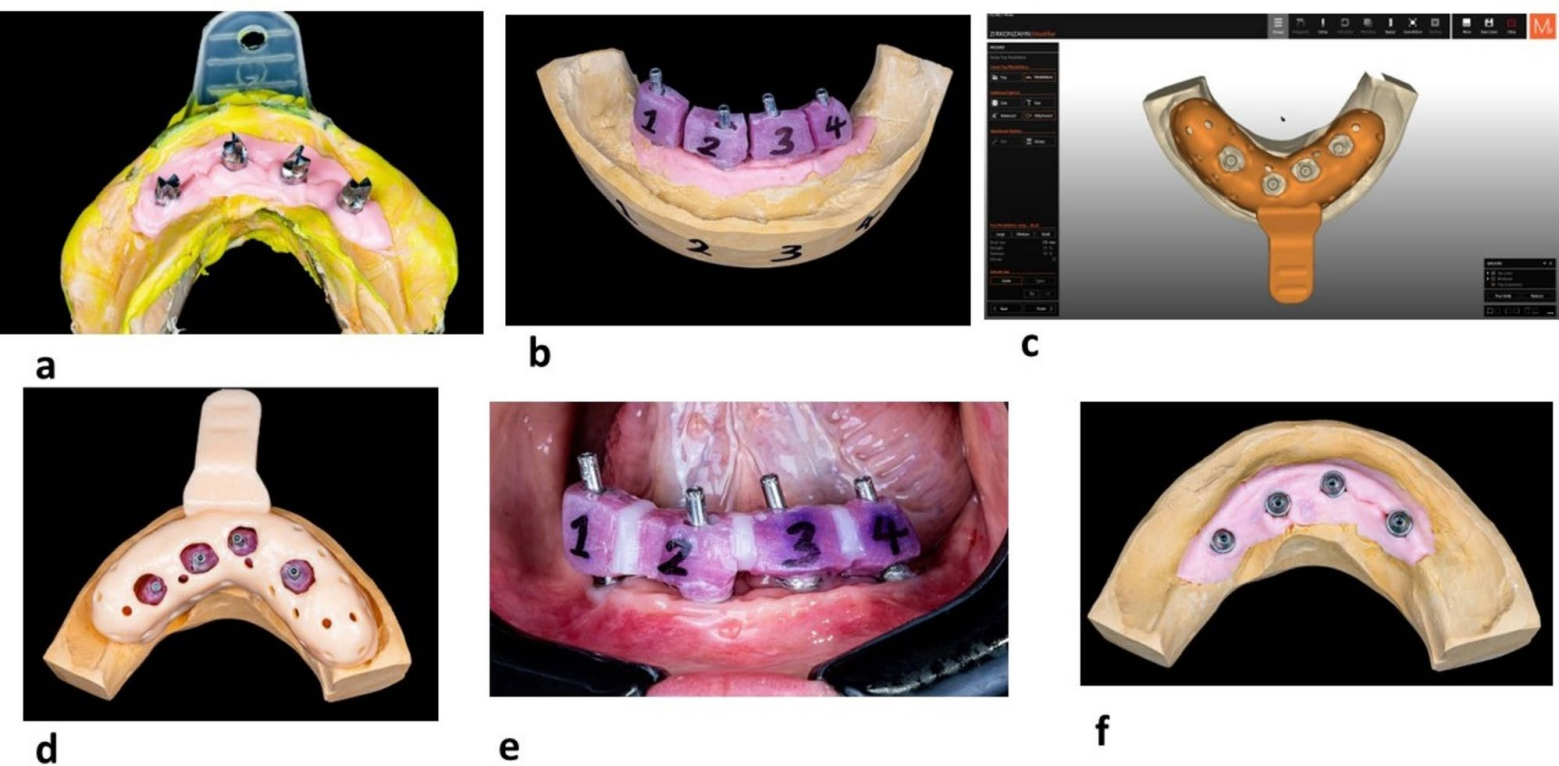
Workflow for constructing the reference cast following the conventional splinted open tray impression technique. **a**. Primary closed tray impression, **b**. cubes of low shrinkage resin material to be splinted intraorally, **c**, **d**. design and 3d printing of the customized open tray **e**, resin cubes are splinted in the patient’s mouth with low shrinkage flowable composite **f**. verified master cast to be used as a reference model

For a verified conventional splinted impression, an open-tray impression technique was performed by attaching the open-tray impression copings (Non-Engaging Multi-unit transfer impression copings, Neobiotech) unsplinted to the multi-unit abutments on the implant fixtures with the copings being picked up directly using polyvinylsiloxane (PVS) (AFFINIS PRECIOUS, Coltene, Switzerland) (Fig. 1a). Type IV dental stone (Siladent, Dr Bohme & Schops GmbH, Germany) was poured into the impression after being mixed with a vacuum mixer.

A light-curing tray material (Megatray, megadent dental produkte GmbH, Germany) was utilized for splinting the impression copings extra-oral on the primary model. Then the resin jigs were sectioned and numbered (Fig. 1b). The two-step approach saves chair-side time and is less messy. A custom impression tray was designed digitally using Zirkonzahn tray software (Zirkohnzahn GmbH, Gais, Italy) and was 3D printed with AccuafabD1s (Shining3D, China) (Fig. 1c, d).

In the 2nd visit, the sectioned (to-be splinted) open tray impression copings were seated in the patient’s mouth. A radiograph was taken to guarantee that they were perfectly seated. Then they were splinted with low shrinkage flowable composite (Gradia^®^ direct LoFlo GC, Japan) (Fig. 1e). A trial fit for the customized impression tray was made to ensure that the pins pass completely through the holes. Adjustments to the position of the holes might be required. Then an open tray impression was taken using Affinis Precious (Coltene, Switzerland). To ensure a consistent impression technique and seating pressure, all impressions were taken by only one operator who had years of experience. Consistent ambient humidity and room temperature were maintained while taking conventional impressions, and all manufacturers’ instructions were followed. Materials were cleaned with immersion disinfection (Eurosept Plus Impression Disinfection Liquid; Henry Schein, Langen, Germany) and subsequently rinsed with water. The definitive cast was poured with Type IV dental stone according to the manufacturer’s instructions (Fig. 1f).

**Fig. 2:**
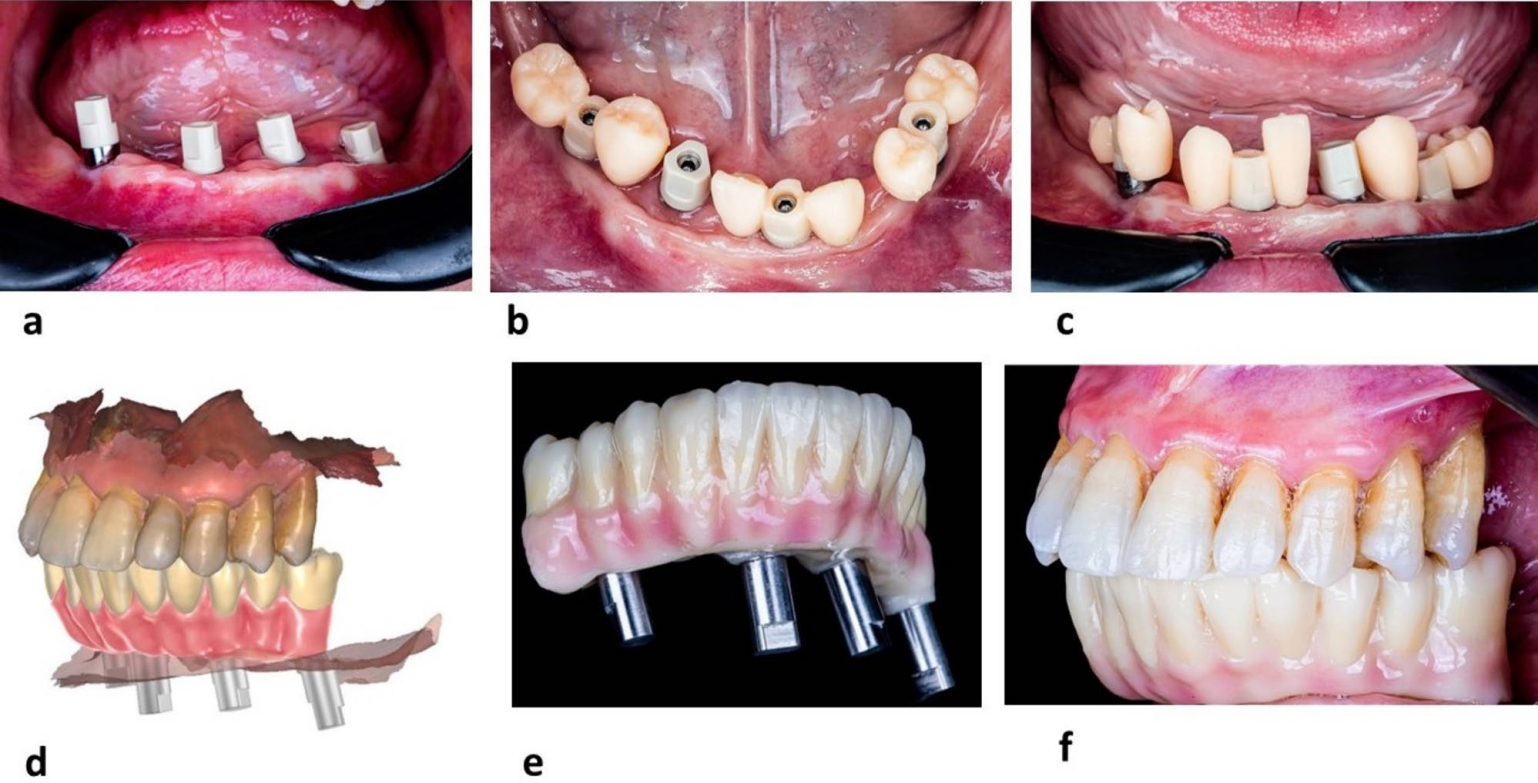
**a**. Digital impression using conventional MUA Scan bodies, **b**, **c**. TMSB showing how tooth geometries act as stable landmarks by Occupying the inter-implant soft tissue areas, **d**. designing of full arch screw retained prosthesis utilizing Exocad software, **e**, **f**. Full arch screw retained monolithic zirconia delivered to the patient

Then, two IOS impressions were recorded with an intra-oral scanner (AoralScan 3, Shining3D), one using non- engaging conventional implant scan bodies secured at the multiunit abutment level (Neobiotech, Seoul, Korea) (Fig. 2a) and another one utilizing non- engaging tooth modified scan bodies (Fig. 2b, c). 7–8 configurations of TMSB with different mesiodistal widths were designed to accommodate various clinical scenarios. Overlapping between TMSB should be avoided to ensure complete seating. To avoid mirroring during the scanning procedure, TMSBs with similar configurations are not preferred. The IOS device was calibrated right before the impression. The scan strategy was consistent for all the procedures following the manufacturer guidelines and starting from the most distal implant scan body on the patient’s left side.

Titanium frameworks based on the conventional master casts were milled to validate the conventional impressions. The frameworks’ mucosal surfaces were intended to have minimal contact with the gingiva in order to prevent any soft tissue resistance throughout the clinical assessment process. To confirm that the conventional casts would be accurate enough to serve as a reference cast, the fit of the frameworks was assessed intra-orally on the subsequent appointment.

Sheffield one screw test in combination with a 40 μm tip explorer (Dental explorer; Weirong Medical), was utilized for the assessment of any gaps between frameworks and abutments. Once every screw had been tightened, a paralleling technique was used to radiographically evaluate fit. If a poor fit was identified, the conventional impressions and subsequent steps would be carried out once more to produce verified master casts which were used to obtain the reference scans.

For each technique, whole chair side time to accomplish the impression (conventional or digital) was calculated.

CAD-CAM implant-supported screw-retained zirconia-based complete-arch FDPs (ISZ-FDPs) was digitally designed onto master cast reference files and delivered to the patient (Fig. 2d-f).

### Data processing and trueness assessment

The master cast was digitalized with a high-resolution laboratory scanner (DS MIX, Shining3D), with an accuracy of < 7 μm (ISO 12836) to achieve a digital master cast standard tessellation language (STL) file used as reference. For each patient three digital files were obtained: One reference scan, one conventional ISB scan, and one TMSB scan. All the STL files were imported to dental CAD software (exocad Dental CAD; exocad), and scan bodies were converted to implant multi-unit ti-base using a digital library. Then, updated STL files were imported to inspection software Mimics (version 2.4; Mimics, Leuven, Belgium) for trueness assessments. The 2 STL files were superimposed with the STL file of the reference cast using the “N-point registration” and “global registration” algorithms. The three-dimensional discrepancy between 2 STL files was evaluated in terms of linear and angular deviation. A central point & central axis of the virtual MUA ti-base were used for deviation measurements. Linear deviations were assessed for each MUA tibase on the three-space axis (X longitudinal, Y lateral and Z vertical) using the center of the ti-base heads for the deviation measurement. The virtual MUA ti-base’s center point and central axis were utilized to measure deviations. The center of the ti-base heads was used to measure the linear deviations for each MUA ti-base along the three-space axes (X longitudinal, Y lateral, and Z vertical).

**Fig. 3:**
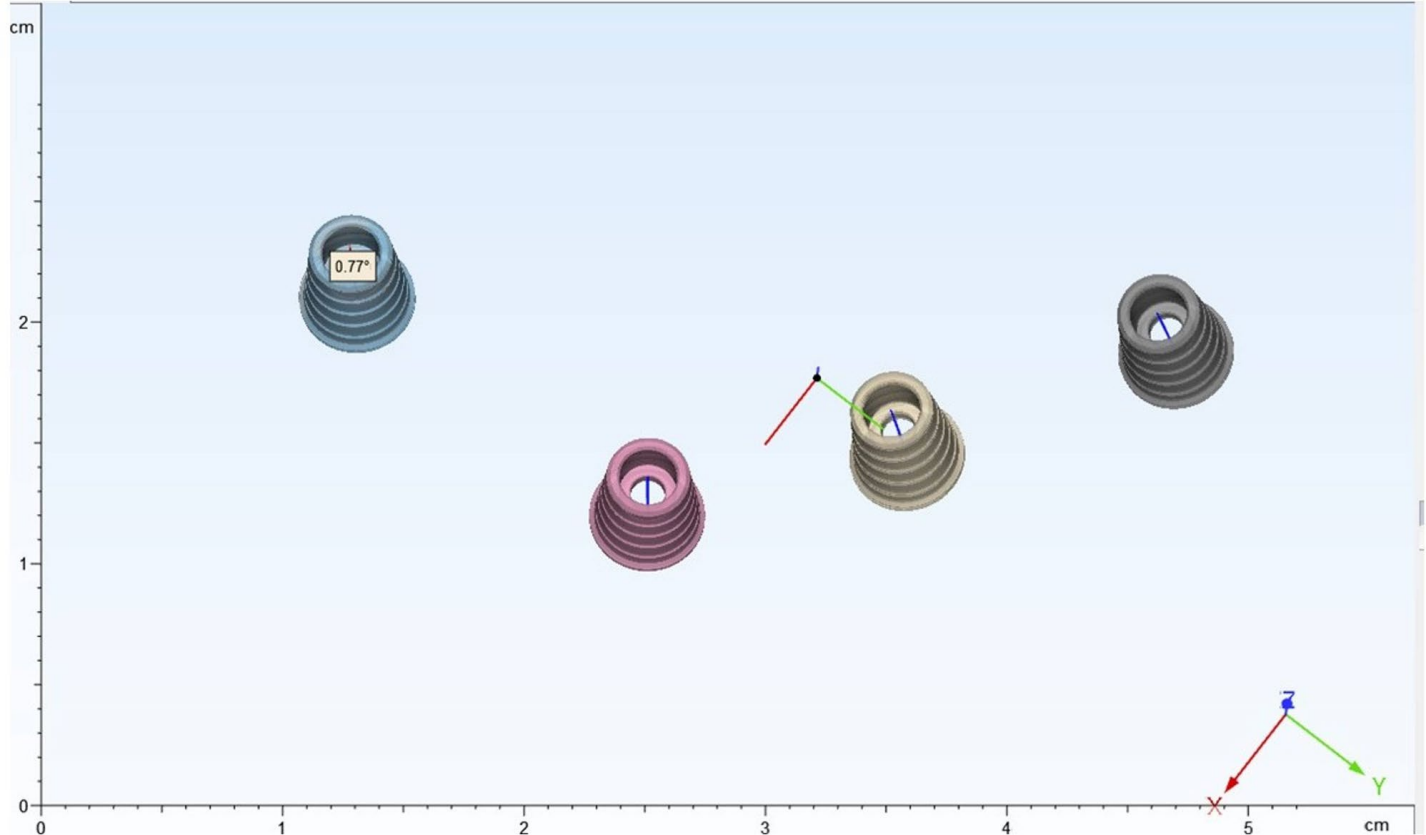
Reference-test scan alignment

**Fig. 4:**
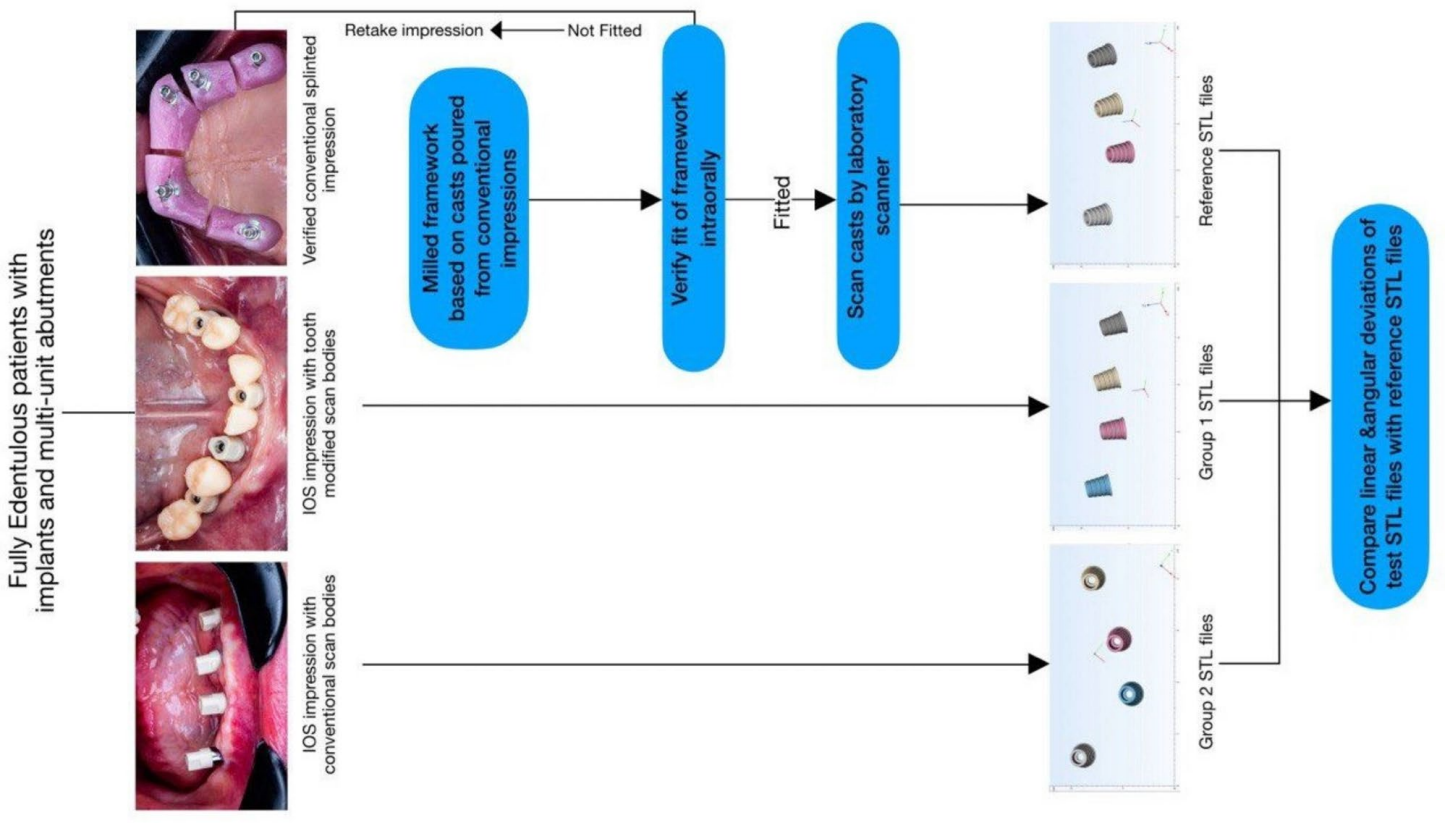
Flow chart explaining the 3 workflows executed in the study

Angular deviations were assessed as the angles formed by the two lines passing perpendicularly through the centers of the test image and the reference image of each ti-base (Fig. 3). To describe the outcome measures concisely, implants in the arch were numbered as implant 1 to implant n (n = number of implants) from the participant’s right to left (Fig. 4).

All statistical analyses were carried out by using a statistical software program (SAS version 9.4; SAS Institute). The linear and angular deviations were analyzed descriptively. The three-dimensional (3D) deviation and the angular deviation were calculated for each implant position and the mean of deviations were obtained in each group (TMSB & CSB) then, statistical significance was evaluated by paired t-test. Both the distance and angular deviations and the clinically acceptable level of misfit (100 μm, 1 degree respectively), considered as the hypothetical mean [21–23], were compared by using a 1-sample t-test. A point-biserial correlation was run to determine the relationship between the type of Jaw and both angular and ΔEUC deviations of both groups (TMSB & CSB). The mean chair side scanning time for the two digital techniques was compared using a paired t-test [24].

## Results

Seven edentulous arches (two maxillae, five mandibles) in 6 patients were rehabilitated with monolithic zirconia screw-retained implant prostheses supported by 4 (*n* = 1) and 5 implants (*n* = 6) for a total amount of 34 implants. Implant positions were scanned by IOS using two types of Scan Bodies (CSB & TMSB) for a total of 68 implant positions recorded to be compared to the relative reference scans. Normality was checked with QQ plots, histograms and the Shapiro–Wilk test. The Shapiro–Wilk test suggests that the data does not significantly deviate from normality.

The ΔEUC deviation Of TMSB group had lower values (M = 61.46, SD = 42.12) than that Of CSB group (M = 97.97, SD = 56.69). A t-test for paired samples showed that this difference was statistically significant (*p* = .005), 95% Confidence interval. The Angular Deviation of TMSC group had lower values (M = 0.85, SD = 0.69) than that Of CSB group (M = 1.3, SD = 1.06) that was statistically significant (*p* = .033), 95% Confidence interval. Table 1 describes the deviations from the reference scans of both TMSB and CSB.

**Table 1:** Descriptive analysis of TMSB and CSB 3D, and angular deviations

	Mean	SD	IQR	Min	Max
**TMSB**					
ΔEUC (µm)	61.46	42.12	47.13	3.3	160
Angle (°)	0.85	0.69	0.94	0.08	2.48
**CSB**					
ΔEUC (µm)	97.97	56.69	89.45	8	230
Angle (°)	1.3	1.06	1.23	0.03	4.99

Abbreviations TMSB, Tooth modified Scan Body; CSB, Conventional scan Body

A one-sample t-test revealed that the overall mean of 3D linear deviation of TMSB Group (M = 61.64, SD = 42.12 μm) was significantly far lower than 100 μm (*P* < .001) that was determined by many studies as the acceptable level of miss fit for full arch digital impressions [21, 25]. On the other hand, CSB group had an overall mean ± standard deviation of 97.97 ± 56.69 μm which was not statistically significant than 100 μm (*P* = .836).

A point-biserial correlation was run to determine the relationship between type of Jaw and both angular and ΔEUC deviations of both groups (TMSB & CSB). There was a correlation between type of Jaw and both angular and ΔEUC deviation of both groups, which was statistically significant. The upper jaws had lower values for both linear and angular deviations when compared with the lower jaws.

**Fig. 5:**
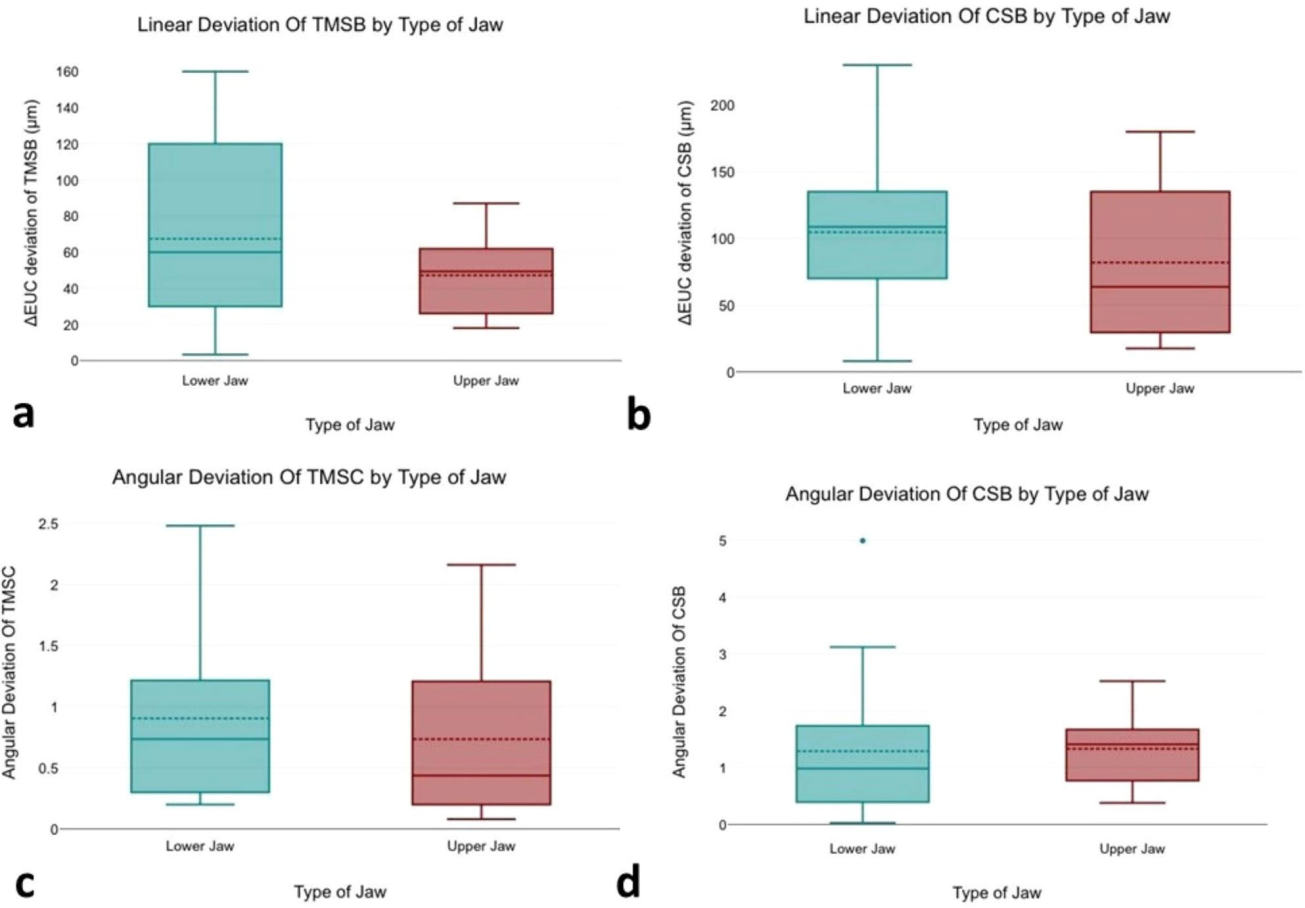
**a**, **b**. Statistical Plot graphs showing a point-biserial correlation between the type of jaw (Maxilla or mandible) and linear deviation when utilizing TMSB and CSB, **c**, **d**. Statistical Plot graphs showing a point-biserial correlation between the type of jaw (Maxilla or mandible) and angular deviation when utilizing TMSB and CSB

Figure 5 shows the correlation between type of arch (maxilla vs. mandible) and both 3D linear and angular deviation for both groups.

The mean time for single jaw digital impression using TMSB was 5.79 min. (SD = 1.44; range 4–8 min.) and 10.86 min. (SD = 1.8; range8-13 min.) for single arch IOS scan using CSB. The meantime gain was 5.07 min or 72.44%. The difference is statistically significant (Table 2).

**Table 2:** The mean time for single for single arch IOS scan using TMSB or CSB

	Time for single arch IOS scan using TMSB (min.)	Time for single arch IOS scan using CSB (min.)	Time gain (min.)
1	4	12.5	8.5
2	5.5	10	4.5
3	6.5	11	4.5
4	8	12	4
5	7	8	1
6	5	9.5	4.5
7	4.5	13	8.5

## Discussion

This clinical trial aimed to investigate intra-oral scanning accuracy for complete-arch implant scanning with a novel scan body geometry, that acts as inter implant stable landmark in addition to its main function as a virtual implant locator. TMSB revealed a significantly lower linear and angular deviation than conventional scan body, so the null hypothesis of the primary endpoint was rejected. The lower arch showed a statistically significant correlation with both linear and angular deviation, so the null hypothesis of the secondary outcome was also rejected.

There are a lot of studies that showed sufficient accuracy obtained when utilizing optical impression in dentate patient for different types of restorations even those with long-span implant superstructures [16, 25, 26]. The main challenge usually faced in full arch implant restorations is due to lack of fixed objects that act as sable landmarks which facilitate stitching during the scanning process and affect the accuracy of the resultant STL file [18].

Previous studies revealed the superiority of splinted conventional impression techniques in gaining a more accurate master cast, and this yielded a passive fit complete-arch, one-piece fixed prostheses [7, 27]. In this study, the splinted technique was utilized for the fabrication of the reference master cast which was used for comparing the trueness of STL files obtained from scanning conventional scan bodies and modified scan bodies. This approach was followed in most of studies that sought reference for investigating the accuracy of new techniques [18, 28].

The accuracy and scan time were examined in 2020 by Mizumoto et al. using the TRIOS (3Shape) IOS in conjunction with four distinct intraoral scan techniques: the unmodified master model (NO), glass fiduciary markers placed on the edentulous ridge (GB), pressure-indicating paste brushed over the palate and ridge (PP), and floss tied between the scan bodies (FL). These modifications act as artificial landmarks that were proposed to enhance accuracy in full arch scanning. Additionally, five distinct commercially available scan body systems were examined: Atlantis I-Flo (AF), Core3D (C3D), Nt-Trading (NT), Dess-USA (DE), and Zimmer Biomet (ZI). All scan bodies and scan techniques resulted in a distance deviation greater than 170 μm and an angular deviation greater than 0.5 degrees. Splinting scan bodies with floss led to significantly more distance deviation. Surface landmarks like fiduciary markers and pressure indicating paste between scan bodies resulted in similar distance deviations as the technique without any modifications [19].

In another in vitro study, 3 artificial landmarks (pressure-indicating paste -PIP, liquid dam markers placed on the edentulous ridge -LD, and floss tied with pattern resin between the scan bodies FL) and 3 scanning patterns (lingual-buccal pattern—LB, S-shaped pattern—SS, and quadrant pattern—QP) were compared for their effect on the accuracy of the complete-arch implant intraoral digital scans using IOS (Trios^®^4, 3Shape) [20]. In contrast to the previous study, LD artificial landmark was found to have the lowest root mean square (RMS) values of trueness for the scanning pattern of SS (56 μm) and QP (45 μm) groups whilst the highest RMS values of trueness was recorded in the CON and FL artificial landmarks (all *P* < .05) [20].

In the present study, the landmark is part of the scan body itself (TMSB) which is attached directly to the implant, so stability is guaranteed. Also, this landmark was configured according to the natural tooth shape which facilitates the process of capturing because the artificial intelligence of all intra-oral scanners was adjusted to scan tooth shapes. Utilization of this stable landmark significantly improves the linear (61 μm vs. 97 μm) and angular deviation (0.85 vs. 1.3) when compared with the conventional scanning technique (CSB). These results were consistent with the previous study and in contrast with Mizumoto study who reveal a non-significant effect of addition of artificial landmarks during full arch scanning. This may be explained by that, full arch scanning of the cast (in vitro study) is completely different than intraoral scanning (in vivo study) [28].

Benefits of utilizing artificial landmarks may not appear in some in vitro studies due to the absence of challenges that are present intraorally like edentulous areas with mobile mucosa, limited mouth opening, movement of the patient, and tongue which can lead to significant scanning and stitching problems. In a recent study that compared accuracy of in intraoral scanning (in vivo) with cast scanning (in vitro) of the same patient, the authors revealed differences that could be of clinical significance (143 ± 51 μm) and they emphasized that the results of in vitro accuracy studies cannot be directly transferred to the clinical field [28].

Determining the clinically acceptable linear and angular deviations is a challenging task, as it relies on various parameters such as the number and orientation of implants, connection type, manufacturing tolerances of the components, and other factors. Again, there is a lack of consensus in the literature regarding acceptable ranges for misfits as well as how to evaluate passive fit. These limits can be established according to the three-dimensional variation of each implant, the distance between them, the individual and relative angulation, etc [28]. The acceptable misfit in the literature varies from 50 to 150 μm [22, 23, 29, 30]. One classical study reported the clinically acceptable misfit to be 150 μm [22]. A recent study by Manzella et al. investigated the degree of tolerable misfit utilizing stone indicating device as a brittle material that is fractured with any degree of intolerable misfit. The indicating device did fracture in cases of 150 μm horizontal misfit, 50 μm vertical misfit, and 1 degree of angular deviation considering these values as the threshold of acceptable misfit [21]. In the present study, both TMSB and CSB showed Euclidean deviation less than 150 μm but only the TMSB group showed mean angular deviation less than 1 degree. When considering 100 μm as a hypothetical mean of the threshold deviation, only TMSB showed statistically lower value indicating a significant effect of this novel scan body in scanning accuracy. To the author’s knowledge, it is the first in vivo study that is used tooth shaped objects attached to scan bodies which act as a stable landmark.

A recent in vivo study comparing a stereophotogrammetry system with two intra oral scanners in full arch implant scanning. The utilization of photogrammetry in the dental field leads to exclusion of intraoral dental and gingival anatomies while scanning the implant coordinates. Also, no need for stitching during full arch implant scanning which is reflected in the trueness and precision of the scanned object [31]. This study considered 75 μm as the threshold linear deviation limit and o.6 degree as the angular deviation limit. The percentage of distances that were below the 75 μm arbitrary limit was for maxillary and mandibular, respectively: 98.6% and 95.4% for PIC system, 90.4% and 80.0% for true definition scanner, and 77.9% and 68.2% for trios 3 scanner. The percentage of angulations below the arbitrary limit of 0.6 degrees were; 100% and 100% for PIC, 91.4% and 81.4% for TD, and 87.1% and 55.7% for T. The authors conclude that, PIC system obtained better accuracy which is not affected by the type of the arch while the both intra oral scanners performed worse in the mandibular arch [25].These results are concordant with the present study which revealed a positive correlation between linear and angular deviation with the lower arch. This makes sense due to lack of sufficient amount of stable mucosa in the lower arch when compared with upper arch.

In the present study, utilization of TMSB affects not only the accuracy of capturing but also the ease of the process itself. This is demonstrated clearly by the statistically significant difference in scanning times when TMSB (5.79 min) and CSB (10.86 min) were utilized.

The limitations of the present study were that no repetitions were conducted, so precision was not among the results and only trueness could be calculated. This was due to the in vivo nature of this study. No standardization of implant number (4 or 5 implants) or type of arch(maxilla or mandible) added to the study limitations. Also, the results of this study were based on only one intra-oral scanning system, so these tooth-modified scan bodies should be investigated with different intra oral scanning technologies. The results of the current study based only on acquisition step and there is no investigation carried on production steps.

## Conclusions

Considering the limitations of this study, intra-oral scanning can be utilized in some full arch cases. Tooth-modified scan body (TMSB) may improve the ease and accuracy of full arch implant scanning.

## Data Availability

The data that support the findings of this study are available from the corresponding author upon reasonable request.
